# Second‐Look Arthroscopy Revealing Cartilage Coverage Following Arthroscopic Synovectomy in Severe Hemophilic Arthropathy of the Knee: A Case Report

**DOI:** 10.1002/ccr3.72630

**Published:** 2026-04-29

**Authors:** Takeshi Seki, Toshiyuki Tateiwa, Yushi Chikasawa, Tsunehito Ishida, Kagehiro Amano, Ei Kinai, Yuichi Shibairi, Ryui Miyashita, Takahiko Okuda, Mitsutaka Hoshi, Toshitaka Nagao, Jun Matsubayashi, Takahisa Haraguchi, Kengo Yamamoto

**Affiliations:** ^1^ Department of Orthopedic Surgery Tokyo Medical University Tokyo Japan; ^2^ Department of Laboratory Medicine Tokyo Medical University Tokyo Japan; ^3^ Department of Anatomic Pathology Tokyo Medical University Tokyo Japan

**Keywords:** 2nd look arthroscopy, arthroscopic synovectomy, cartilage coverage, hemophilia B, hemophilic arthropathy, knee joint

## Abstract

Arthroscopic synovectomy for severe hemophilic knee arthropathy revealed previously unrecognized microbleeding. Although a second surgery was required because of catching symptoms, cartilage coverage was confirmed, no recurrent bleeding occurred, and clinical symptoms improved.

## Graphical abstract

Arthroscopic findings during the initial surgery in a patient with advanced hemophilic knee arthropathy. Villous synovial proliferation with hemosiderin deposition and exposed subchondral bone were observed, along with pinpoint bleeding on the lateral tibial plateau. Synovectomy resulted in marked reduction of synovial tissue.

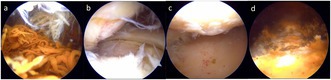

## 1 Introduction

Hemophilia is a hemorrhagic disorder characterized by a deficiency in coagulation factor VIII or IX, leading to a bleeding tendency. In severe cases, patients often suffer from spontaneous recurrent intra‐articular bleeding [1]. The knee joint is one of the most affected sites. Repeated bleeding into the joint leads to synovitis and progressive degeneration of the articular cartilage, ultimately resulting in hemophilic arthropathy [2]. This condition causes pain, limited range of motion, and impaired activities of daily living, significantly reducing quality of life (QOL), even in young individuals. Prophylactic replacement therapy using coagulation factor concentrates is the standard for preventing and controlling intra‐articular bleeding. Arthroscopic synovectomy has been introduced as an early intervention targeting the synovium, aiming to physically remove the bleeding source, reduce intra‐articular bleeding, alleviate pain, and improve joint function.

However, the postoperative changes in joint structure—particularly regarding articular cartilage—remain unclear, and there are no definitive reports demonstrating cartilage repair following arthroscopic synovectomy alone. In cases with advanced joint degeneration, the degenerative changes are generally considered irreversible, and radiographic progression has been reported even after surgery [3, 4, 5].

We report a case of hemophilic arthropathy in a young adult male with severe hemophilia B classified as Arnold‐Hilgartner Stage IV, who underwent arthroscopic synovectomy. Although reoperation was required 19 months after the initial surgery due to catching symptoms, we observed clinical improvements in joint range of motion (ROM) and functional scores, and notably, MRI and second‐look arthroscopy revealed cartilage coverage, a finding that has not been previously described in the literature [6]. This case may provide new insights into intra‐articular environmental changes and potential cartilage coverage following synovectomy in hemophilic arthropathy.

## 2 Case History and Examination

The patient was a 38‐year‐old man with a history of severe hemophilia B who presented with right knee pain. He had experienced knee pain since his teenage years and had been followed at an internal medicine outpatient clinic. From the age of 30, his symptoms gradually worsened, and over the past 8 years, he had experienced four episodes of symptomatic hemarthrosis; however, no episodes were noted in the year prior to presentation. As his knee pain began to significantly interfere with his work in retail, he was referred for orthopedic evaluation.

He had a normal body habitus, measuring 164.6 cm in height and 51.5 kg in weight (BMI 19.01 kg/m^2^). The right knee showed marked suprapatellar swelling. ROM was limited to 135° flexion and −10° extension. There was marked muscle atrophy with a 7.5 cm difference in thigh circumference between the limbs.

## 3 Investigation and Treatment

**FIGURE 1:**
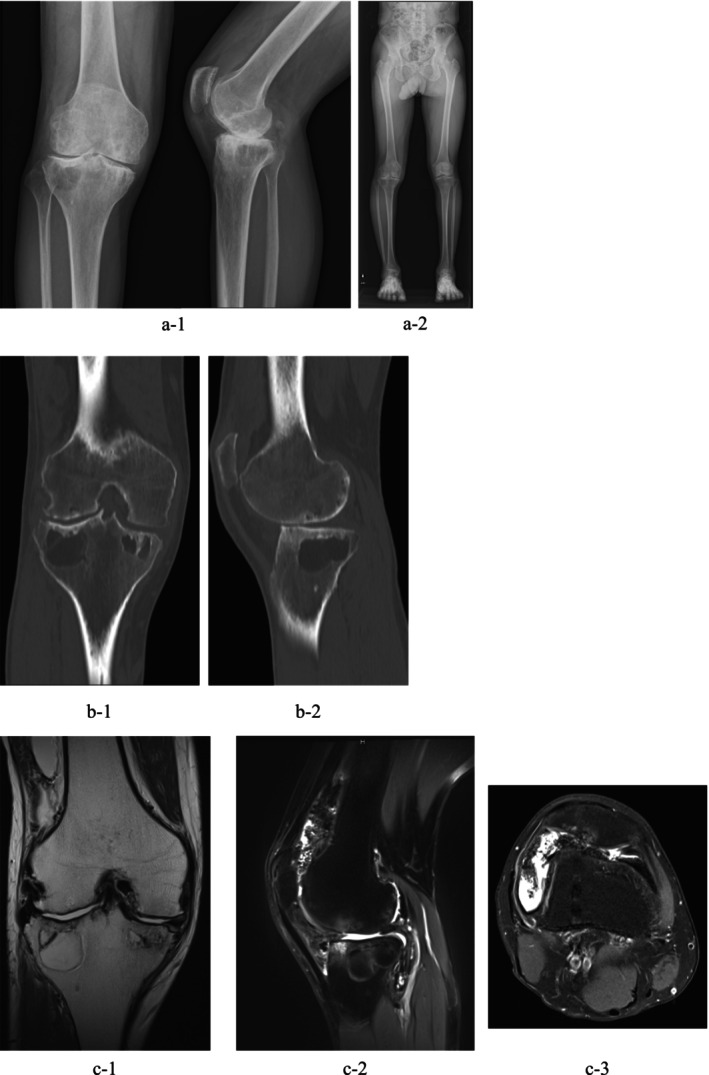
Preoperative imaging findings before initial surgery. (a‐1) Anteroposterior and lateral plain radiographs of the right knee. (a‐2) Full‐length standing radiograph showing overall lower limb alignment. Plain CT images (b‐1)coronal views, (b‐2)sagittal views. MRI of the right knee: (c‐1) Proton density‐weighted image (coronal section)(c‐2) Proton density fat‐suppressed image (sagittal section)(c‐3) Proton density fat‐suppressed image (axial section).

Plain radiographs revealed joint space narrowing in both medial and lateral compartments and multiple large subchondral cysts beneath the tibial articular surface, without destructive changes of the joint surface. These findings were consistent with Arnold‐Hilgartner Stage IV arthropathy (Figure 1a,b).

Lower limb alignment showed:

- FTA (femorotibial angle): 177°.
- MPTA (medial proximal tibial angle): 83°.
- mLDFA (mechanical lateral distal femoral angle): 86°.
- HKA (hip‐knee‐ankle angle): 178°.
- JLO (joint line obliquity): 169°—with no significant varus or valgus deformity.

MRI showed joint effusion in the suprapatellar pouch, along with villous synovial hypertrophy and suspected hemosiderin deposition. The articular cartilage of both medial and lateral compartments was significantly thinned (Figure 1c). Conservative pain management with a knee brace was ineffective, and arthroscopic synovectomy was performed.

**FIGURE 2:**
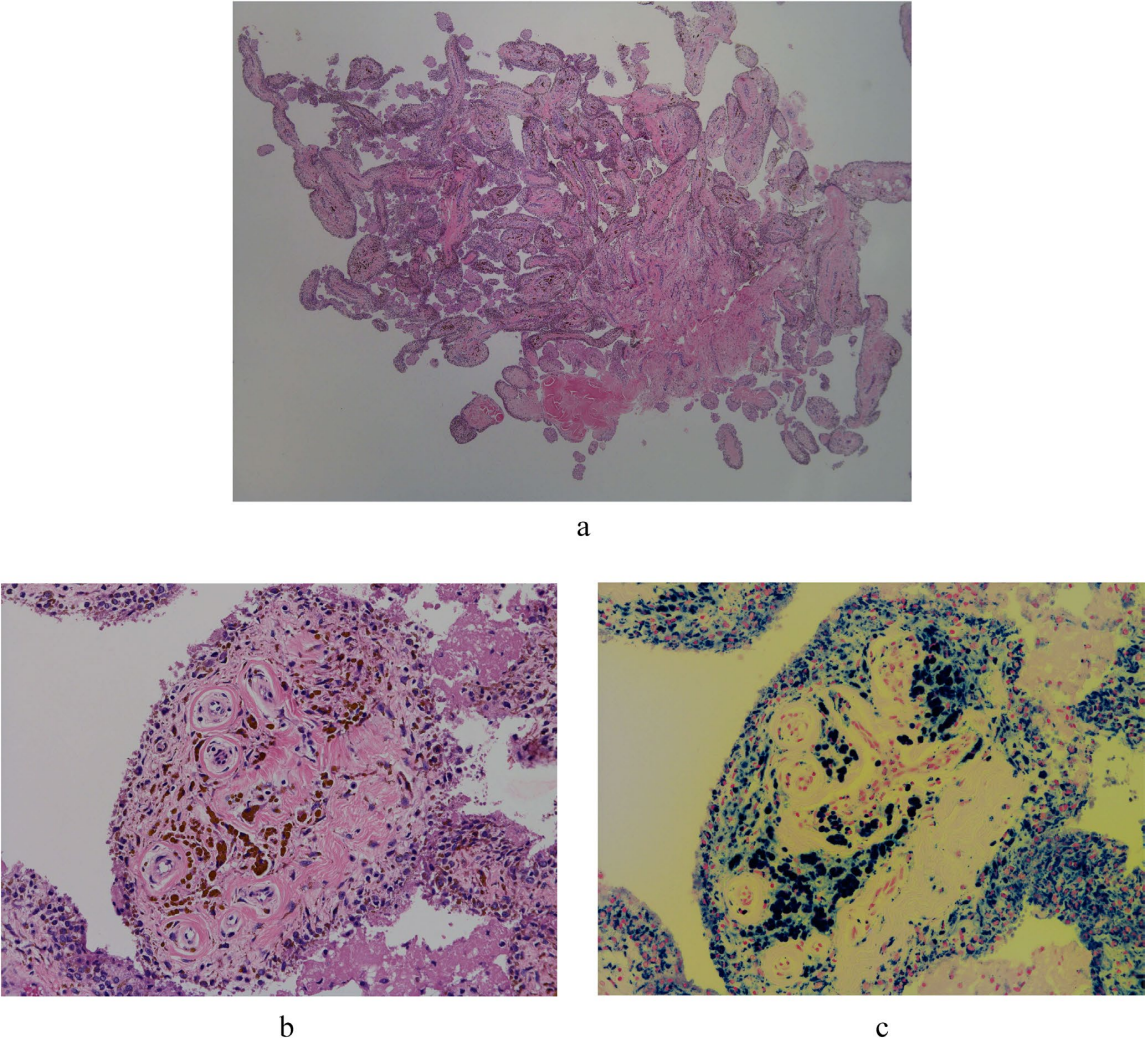
Pathological findings of the synovial membrane during knee arthroscopy. (a) HE stain, low magnification: The synovial membrane is edematous and proliferative. (b) HE‐stained high‐power field view: Brown cells are deposited in synovial cells and the sub‐synovial layer, and clusters of histiocytes phagocytosing brown cells are present in the sub‐synovial layer. (c) Iron‐stained high‐power field view: Brown cells are stained by iron staining and show hemosiderin deposition.

**FIGURE 3:**
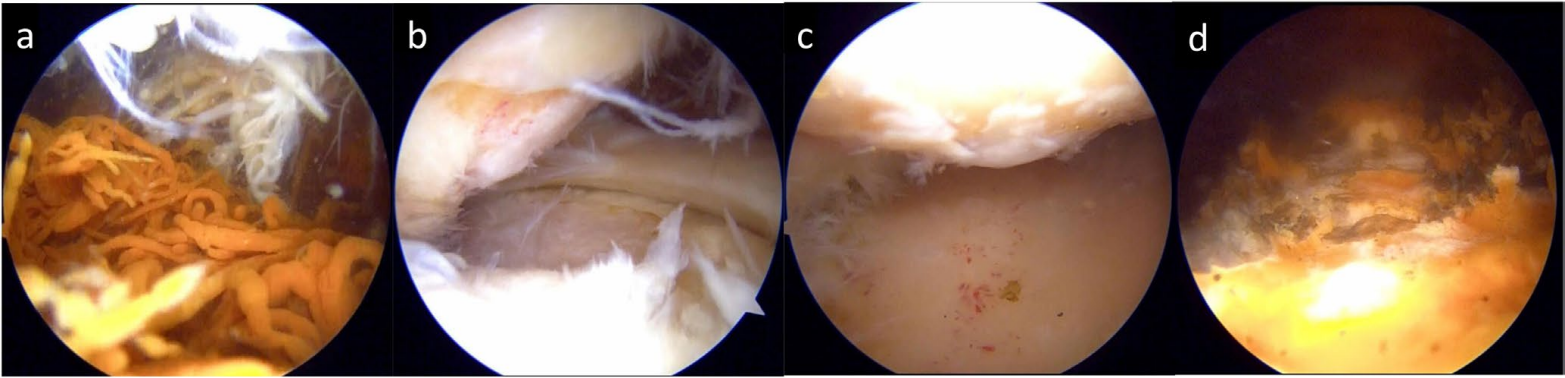
Arthroscopic findings during the initial surgery. (a) Suprapatellar pouch showing villous synovial proliferation with hemosiderin deposition. (b) Medial femoral condyle with exposed subchondral bone (ICRS Grade IV). (c) Lateral tibial plateau showing pinpoint bleeding and cartilage loss. (d) Appearance of the suprapatellar pouch after synovectomy.

The procedure was performed without a tourniquet, with a bolus administration of 3000 U of recombinant eftrenonacog alfa. A radiofrequency device was primarily used for synovectomy rather than a shaver. The suprapatellar pouch showed pronounced villous synovitis with hemosiderin deposition and was highly prone to bleeding (Figures 2, 3a). On arthroscopy, about half of the medial femoral condyle had exposed subchondral bone corresponding to ICRS Grade IV cartilage damage (Figure 3b) [7]. The lateral tibial plateau also showed diffuse subchondral bone exposure with punctate bleeding, which the patient had not recognized (Figure 3c). Because adequate hemostasis was achieved during synovectomy, no postoperative drain was inserted (Figure 3d). Weight‐bearing and ROM exercises were initiated the day after surgery according to pain tolerance. Recombinant eftrenonacog alfa was administered as a bolus every 24 h during hospitalization. After the swelling had improved and gait had stabilized, the patient was discharged home on postoperative day 10.

## 4 Outcome and Follow‐Up

**FIGURE 4:**
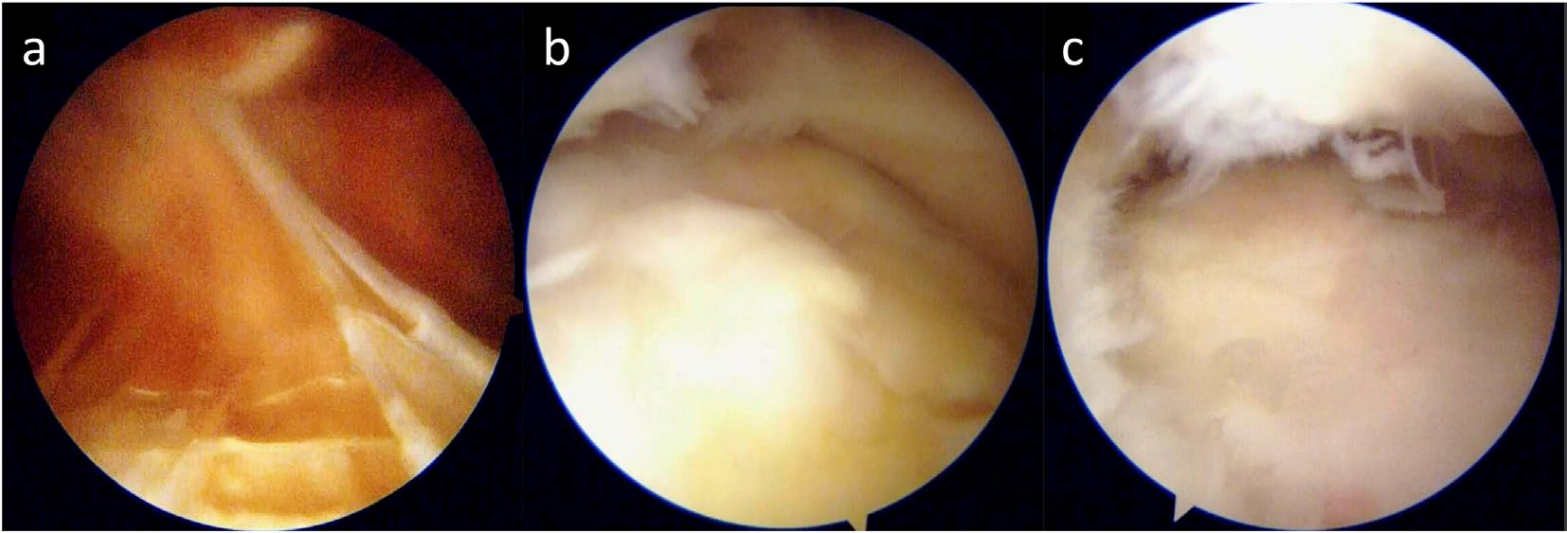
Arthroscopic findings at second‐look surgery (19 months postoperatively). (a) Suprapatellar pouch showing minimal synovial regrowth. (b) Medial femoral condyle covered by white, fibrocartilage‐like tissue. (c) Lateral tibial plateau showing no subchondral bone exposure.

**FIGURE 5:**
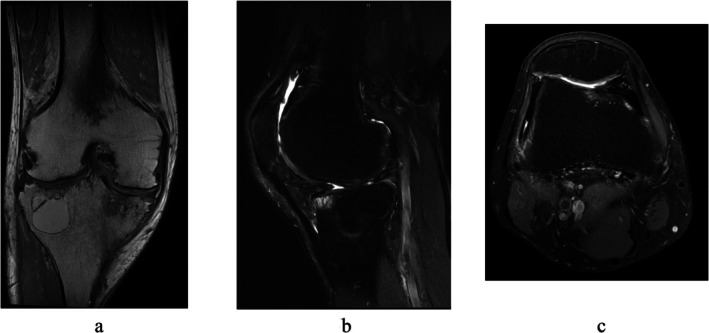
MRI before the second look surgery. (a) Proton density‐weighted image (coronal section). (b) Proton density fat‐suppressed image (sagittal section). (c) Proton density fat‐suppressed image (horizontal section).

The patient experienced gradual pain relief and no postoperative bleeding episodes. ROM normalized, and clinical scores improved. However, 19 months after the initial surgery, the patient began to experience catching symptoms during daily activities without any apparent trigger, prompting a second arthroscopic surgery (Figure 4). MRI prior to reoperation showed that the villous synovium of the suprapatellar pouch had disappeared and a new layer of cartilage had formed on the weight‐bearing surface (Figure 5).

The procedure was performed without a tourniquet, with a bolus administration of 3000 U of recombinant nonacog alfa. Intraoperatively, impingement was observed due to scar tissue around the ACL and intercondylar notch, which was resected using a shaver. Synovial and cartilage evaluation showed that suprapatellar synovial hypertrophy had almost completely resolved (Figure 4a). The articular surfaces of the femur and tibia were covered with white fibrocartilage‐like tissue, and no subchondral bone was exposed (Figure 4b,c).

**FIGURE 6:**
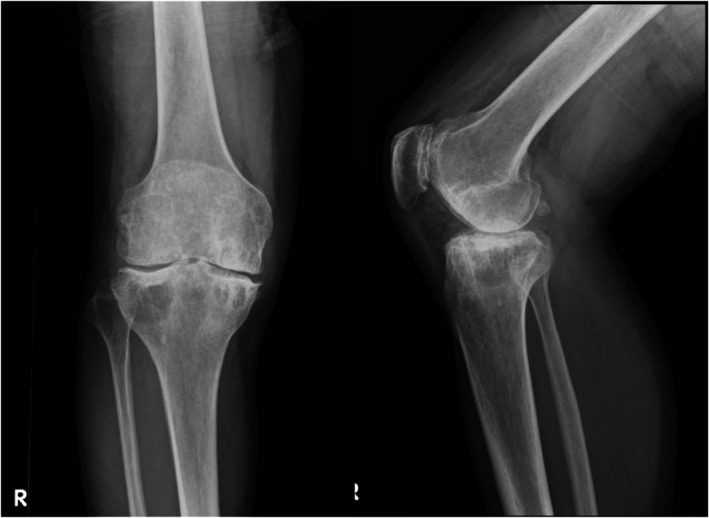
Plain radiographs 19 months after the second surgery. Anteroposterior and lateral views showing no progression of radiographic joint destruction.

As with the initial procedure, no drain was inserted postoperatively, and weight‐bearing and ROM training were resumed the following day based on pain. Recombinant eftrenonacog alfa was administered as a bolus every 24 h until discharge. After the swelling had subsided and gait had stabilized, the patient was discharged home on postoperative day 4. At 18 months following the second surgery, the patient's knee pain had further improved compared to the time of reoperation. There were no episodes of joint bleeding, and clinical scores continued to improve (Table 1). No radiographic progression of arthropathy was observed on follow‐up imaging (Figure 6).

**TABLE 1:** Detailed summary of previous reports on arthroscopic surgery for hemophilia of the knee.

	Before initial surgery	19 months after initial surgery	18 months after reoperation
Flexion ROM (Right/Left) (°)	135/140	140/140	140/140
Extension ROM (Right/Left) (°)	−10/0	0/0	0/0
Tigh circumference (Right/Left) (cm)	34.5/42.0	39.0/43.0	40.0/44.0
Maximum circumference of lower leg (Right/Left) (cm)	30.5/32.0	32.0/32.5	32.0/32.5
JOA score	60	85	85
Lysholm score	41	63	73
IKDC subjective score	27.6	39.1	58.6
HJHS total score	37	32	30

*Note:* Details include author, publication year, number of joints treated, mean patient age, follow‐up duration, preoperative staging, and postoperative outcomes.

Abbreviations: HJHS score, Hemophilia Joint Health Score; IKDC score, International Knee Documentation Committee Subjective score; JOA score, Japanese Orthopedics Association score; WOMAC (Western Ontario and McMaster Universities Osteoarthritis Index).

## 5 Discussion

Several noteworthy aspects were observed in the clinical course of this case:

1. Microscopic intra‐articular bleeding, unrecognized by the patient, was directly observed during arthroscopy.
2. Cartilage coverage responses were confirmed by both MRI and second‐look arthroscopy after the initial synovectomy.
3. Although the patient had Arnold‐Hilgartner Stage IV hemophilic arthropathy, arthroscopic synovectomy resulted in improvement in joint ROM and functional scores, even though reoperation was required at 19 months postoperatively.

### 5.1 Subclinical Bleeding and Synovial Hemosiderin Deposition

Hemophilia B is an X‐linked congenital bleeding disorder caused by a deficiency of coagulation factor IX, resulting in an increased tendency to bleed. Patients with severe hemophilia (factor activity < 1%) may experience spontaneous bleeding. In hemarthrosis, hemoglobin derived from red blood cells is taken up by synovial tissue, and its degradation product, hemosiderin, accumulates within the synovium. Synovial tissue containing hemosiderin promotes inflammation and the production of proinflammatory cytokines such as interleukin (IL)‐1, IL‐6, and tumor necrosis factor‐α (TNF‐α), which accelerate secondary degeneration of articular cartilage [8, 9]. These cytokines stimulate matrix metalloproteinases and other catabolic pathways in chondrocytes, leading to progressive cartilage degeneration. In addition, excess iron derived from repeated hemarthrosis can induce oxidative stress within the joint through the generation of reactive oxygen species, a process known as **iron‐induced oxidative stress**, which further impairs chondrocyte function and extracellular matrix homeostasis [9]. Arthroscopic synovectomy removes hypertrophic synovium containing hemosiderin deposits and may reduce the intra‐articular inflammatory burden by eliminating inflamed synovial tissue and decreasing the local production of inflammatory cytokines. As a result, this procedure may contribute to restoring a more favorable intra‐articular environment for chondrocyte metabolism and cartilage homeostasis.

Interestingly, even in the absence of reported bleeding episodes, some patients experience cartilage degeneration due to asymptomatic hemarthrosis [10]. MRI may reveal hemosiderin deposition in joints that appear clinically silent [11, 12]. In this case, despite the absence of hemarthrosis episodes in the previous year, MRI showed marked synovial proliferation and hemosiderin deposition in the suprapatellar pouch, suggesting subclinical bleeding. During surgery, microscopic bleeding was observed on the lateral tibial plateau even under bolus administration of recombinant eftrenonacog alfa. Such bleeding is not detectable on imaging, highlighting the value of arthroscopic evaluation.

### 5.2 Cartilage Repair and Progression of Degeneration After Arthroscopic Synovectomy

Furthermore, although there was no change in the Arnold–Hilgartner classification after the initial surgery, MRI demonstrated coverage of the previously exposed subchondral bone, and second‐look arthroscopy revealed that the area was covered with fibrocartilage‐like tissue. Generally, once cartilage is damaged, spontaneous healing is difficult. Surgical cartilage repair typically requires direct intervention at the subchondral level, such as microfracture, osteochondral autograft transplantation (OATS), or autologous chondrocyte implantation (ACI) [13, 14, 15].

Arthroscopic synovectomy for hemophilic arthropathy has been reported to reduce the frequency of intra‐articular bleeding [3, 16, 17, 18, 19, 20, 21, 22]. In addition, functional outcomes after this procedure are generally considered favorable (Table 2) [4, 5, 22, 23, 24]. However, synovectomy alone is not expected to promote cartilage coverage, and previous studies have mainly described only a delay in the progression of joint degeneration [3, 4, 5]. Buda et al. reported improvement of cartilage in all five cases of hemophilic ankle arthropathy treated with a combination of synovectomy and bone marrow–derived cell transplantation [25]. However, to our knowledge, cartilage coverage following synovectomy alone has not been previously reported. The present case is the first to describe such a finding. Given the rarity of this outcome, such favorable cartilage responses may be limited to highly selected cases.

**TABLE 2:** Reports on functional outcome scores before and after arthroscopic synovectomy for hemophilic knee arthropathy.

Author (Ref.)	Publication year	Number of patients (Knees)	Hemophilia type	Age (years)	Follow‐up duration	Functional score	Outcomes
Yoon et al. [22]	2005	26 (28)	A: 25, B: 1 (Severe: 8, moderate: 17, mild: 3)	17.8 (8–37)	71 M	・The average HSS	Improved
Almeida et al. [23]	2015	8 (9)	A: 8 (Severe: 8)	16.1 (9.6–25)	5Y	IKDC score WOMAC SF‐36 overall score SF‐36 (physical component) SF‐36 (mental component)	Improved Improved Improved Improved No change
Wu et al. [5]	2016	5 (5)	A: 4, B: 1	26.0 (23–28)	30.2	VAS Lysholm score	Improved Improved
Rodriguez‐Merchan et al. [24]	2016	27 (27)	A: 27	28.6 (26–39)	7.5Y	Knee Society pain scores Function scores	Improved Improved
Zhang et al. [4]	2018	11 (11)	A: 8 (Severe 8), B: 3 (Severe 3)	18.7 (12–34)	71.9 M	The mean HSS score The mean pain score	Improved Improved

*Note:* This table summarizes the author, publication year, number of joints treated, mean patient age, follow‐up duration, and pre‐ and postoperative functional scores reported in previous studies.

Abbreviations: HJHS, Hemophilia Joint Health Score; IKDC score, International Knee Documentation Committee subjective score; WOMAC, Western Ontario and McMaster Universities Osteoarthritis Index.

In contrast to relatively favorable functional outcomes, several studies have reported that radiographic joint degeneration may continue to progress after arthroscopic synovectomy (Table 3) [3, 4, 17, 19, 22, 23, 26, 27]. Zhang et al. reported that among nine patients with preoperative Arnold–Hilgartner stage IV arthropathy who underwent arthroscopic synovectomy, two progressed to Arnold–Hilgartner stage V after a mean follow‐up of 71.9 months [4]. In contrast, Yoon et al. reported that among 28 patients who underwent arthroscopic synovectomy and were followed for a mean of 71 months, six had Arnold–Hilgartner stage IV arthropathy preoperatively, and none showed progression of radiographic stage during follow‐up [22]. In the present case, which involves an adult patient with Arnold–Hilgartner stage IV arthropathy, the risk of long‐term radiographic progression may still remain despite the favorable clinical course observed so far.

**TABLE 3:** Reports on pre‐ and postoperative Arnold–Hilgartner radiographic stage in patients undergoing arthroscopic synovectomy for hemophilic knee arthropathy.

Author (Ref.)	Year	Number of patients (Knees)	Hemophilia type	Age (years)	Follow‐up duration	Arnold‐Hilgartner radiographic stage (pre‐operation)	Arnold‐Hilgartner radiographic stage (last follow‐up)
Kim et al. [3]	1984	6 (7)	A: 6 (Severe: 6)	21 (12–31)	50 M	II: 1 III: 2 IV: 4	II‐1 III‐0 IV‐6
Limberd [17]	1987	5 (5)	A: 4, B: 1 (Severe: 5)	20 (10–35)	31 M (24‐38 M)	III: 4 IV: 1	III: 3 IV: 2
Triantafyllou et al. [19]	1992	5 (5)	A: 5	18 (6–31)	26 M	II: 2 III: 3	IV: 4 V: 1
Wiedel et al. [26, 27]	1990, 1996	9 (5)	A: 8, B: 1	16 (8–36)	10‐15Y	II: 3 III: 3 IV: 3	III: 2 IV: 4 V: 2 unkown: 1
Dunn et al. [21]	2004	7 (7)	—	10.0 (4.3–18.4)	11 Y (*n* = 4)	IV: 1 Unkown: 6	IV: 1 Unkown: 6
Yoon et al. [22]	2005	26 (28)	A: 25, B: 1 (Severe: 8, moderate: 17, mild: 3)	17.8 (8–37)	71 M	・No roentgenographic progression was observed in 25/28 cases. II: 4 III: 18 IV: 6	II: 3 III: 17 IV: 8
Almeida et al. [23]	2015	8 (9)	A: 8 (Severe: 8)	16.1 (9.6–25)	5 Y	Average of stage III (II‐IV)	Average of stage IV (III, IV)
Zhang et al. [4]	2018	11 (11)	A: 8, B: 3 (Severe 11)	18.7 (12–34)	71.9 M	IV: 11	IV: 9 V: 2

*Note:* This table summarizes the author, publication year, number of treated joints, mean patient age, follow‐up duration, and the pre‐ and postoperative Arnold–Hilgartner radiographic stage reported in previous studies.

### 5.3 Influence of Joint Stability and Lower Limb Alignment

Other general factors that may influence degenerative changes in the knee regardless of hemophilia include joint instability, lower limb alignment, and body weight [28]. Knee stability is maintained by four major ligaments: the medial and lateral collateral ligaments (MCL, LCL), and the anterior and posterior cruciate ligaments (ACL, PCL). Instability increases abnormal contact stress gradients, which can promote cartilage degradation [29, 30]. At the cellular level, abnormal mechanical loading impairs chondrocyte function and extracellular matrix integrity, promoting cartilage breakdown [29].

Lower limb alignment influences load distribution on the knee. The mechanical axis runs from the femoral head center to the ankle center, and the hip–knee–ankle (HKA) angle between femoral and tibial mechanical axes is normally 180° ± 3° in Japanese individuals [31]. The femorotibial angle (FTA), defined by anatomical axes, averages 175.7° ± 6.5° [32]. Varus and valgus alignments increase the risk of medial and lateral compartment osteoarthritis, respectively [33].

In this case, the patient had a standard body habitus and no joint instability. Although the MPTA was 83°, indicating mild varus alignment, the FTA and HKA indicated that joint stability was preserved. In hemophilic arthropathy, assessing both disease stage and mechanical alignment may help predict postoperative progression and outcomes.

### 5.4 Limitations

Several limitations should be considered when interpreting this case. First, this report describes a single case, and therefore the findings cannot be generalized to all patients with hemophilic arthropathy. Second, the follow‐up period of 37 months from the initial surgery may be insufficient to fully evaluate the long‐term progression of joint degeneration. Third, histological evaluation of the tissue covering the subchondral bone was not performed because the lesion was located on the weight‐bearing articular surface; therefore, it remains unclear whether the observed tissue represented fibrocartilage formation or reparative fibrous tissue rather than true hyaline cartilage regeneration. Furthermore, quantitative MRI assessment of cartilage thickness was not performed, and no biochemical markers of cartilage metabolism or joint inflammation were analyzed. Future studies with larger cohorts, longer follow‐up, and objective imaging or biochemical assessments will be necessary to better clarify the structural changes observed after arthroscopic synovectomy in hemophilic arthropathy.

## 6 Conclusion

Arthroscopic synovectomy in a patient with Arnold‐Hilgartner Stage IV hemophilic arthropathy led to improved joint range of motion, reduced bleeding episodes, and better functional outcomes, despite the need for reoperation at 19 months. Microscopic joint bleeding was observed intraoperatively, and cartilage coverage was confirmed via MRI and second‐look arthroscopy. Assessment of not only the preoperative Arnold‐Hilgartner classification but also joint stability and lower limb alignment may help predict postoperative progression and outcomes after arthroscopic intervention.

## Author Contributions

**Takeshi Seki:** conceptualization, data curation, formal analysis, writing – original draft, writing – review and editing. **Toshiyuki Tateiwa:** writing – review and editing. **Yushi Chikasawa:** investigation, supervision, validation, writing – review and editing. **Tsunehito Ishida:** writing – review and editing. **Kagehiro Amano:** resources, supervision, writing – review and editing. **Ei Kinai:** supervision, writing – review and editing. **Yuichi Shibairi:** writing – review and editing. **Ryui Miyashita:** investigation, writing – review and editing. **Takahiko Okuda:** writing – review and editing. **Mitsutaka Hoshi:** writing – review and editing. **Toshitaka Nagao:** visualization. **Jun Matsubayashi:** visualization. **Takahisa Haraguchi:** investigation, resources. **Kengo Yamamoto:** supervision, writing – review and editing.

## Funding

The authors have nothing to report.

## Consent

The patient provided written informed consent for the publication of clinical data and images for research and educational purposes.

## Conflicts of Interest

The authors declare no conflicts of interest.

## Data Availability

Data available on request from the authors. The data that support the findings of this study are available from the corresponding author upon reasonable request.

## References

[1] P. M. Mannucci and E. G. Tuddenham , “The Hemophilias—From Royal Genes to Gene Therapy,” New England Journal of Medicine 344, no. 23 (2001): 1773–1779, 10.1056/NEJM200106073442307. 11396445

[2] L. A. Valentino , “Blood‐Induced Joint Disease: The Pathophysiology of Hemophilic Arthropathy,” Journal of Thrombosis and Haemostasis 8, no. 9 (2010): 1895–1902, 10.1111/j.1538-7836.2010.03962.x. 20586922

[3] H. C. Kim , K. Klein , S. Hirsch , J. R. Seibold , J. Eisele , and P. Saidi , “Arthroscopic Synovectomy in the Treatment of Hemophilic Synovitis,” Scandinavian Journal of Haematology. Supplementum 40 (1984): 271–279, 10.1111/j.1600-0609.1984.tb02573.x. 6591394

[4] T. Zhang , S. Huang , S. Xu , H. Li , X. He , and F. Zhang , “Clinical Outcomes of Arthroscopic Synovectomy for Adolescent or Young Adult Patients With Advanced Haemophilic Arthropathy,” Experimental and Therapeutic Medicine 16, no. 5 (2018): 3883–3888, 10.3892/etm.2018.6709. 30344665 PMC6176150

[5] L. T. Wu , H. T. Lu , C. H. Chen , A. Ko , and C. H. Lee , “Arthroscopic Synovectomy Considerably Reduces Bleeding Frequency and Improves Joint Function in Hemophilic Patients With Chronic Synovitis,” Formosan Journal of Surgery 49, no. 2 (2016): 49–55.

[6] W. D. Arnold and M. W. Hilgartner , “Hemophilic Arthropathy. Current Concepts of Pathogenesis and Management,” Journal of Bone and Joint Surgery. American Volume 59, no. 3 (1977): 287–305. 849938

[7] M. Brittberg and C. S. Winalski , “Evaluation of Cartilage Injuries and Repair,” Journal of Bone and Joint Surgery. American Volume 85, no. 2 (2003): 58–69, 10.2106/00004623-200300002-00008. 12721346

[8] N. W. Jansen , G. Roosendaal , and F. P. Lafeber , “Understanding Haemophilic Arthropathy: An Exploration of Current Open Issues,” British Journal of Haematology 143, no. 5 (2008): 632–640, 10.1111/j.1365-2141.2008.07386.x. 18950457

[9] G. Roosendaal and F. P. Lafeber , “Blood‐Induced Joint Damage in Hemophilia,” Seminars in Thrombosis and Hemostasis 29, no. 1 (2003): 37–42, 10.1055/s-2003-37938. 12640563

[10] C. Daffunchio , G. Galatro , V. Faurlin , D. Neme , and H. Caviglia , “The Hidden Joint in Children With Haemophilia on Prophylaxis,” Thrombosis Research 226 (2023): 86–92, 10.1016/j.thromres.2023.04.012. 37130495

[11] R. Gooding , J. Thachil , J. Alamelu , J. Motwani , and P. Chowdary , “Asymptomatic Joint Bleeding and Joint Health in Hemophilia: A Review of Variables, Methods, and Biomarkers,” Journal of Blood Medicine 12, no. 1 (2021): 209–220, 10.2147/JBM.S304597. 33833602 PMC8023018

[12] F. H. P. van Leeuwen , E. D. P. van Bergen , M. A. Timmer , et al., “Magnetic Resonance Imaging Evidence for Subclinical Joint Bleeding in a Dutch Population of People With Severe Hemophilia on Prophylaxis,” Journal of Thrombosis and Haemostasis 21, no. 5 (2023): 1156–1163, 10.1016/j.jtha.2023.01.035. 36758725

[13] J. R. Steadman , W. G. Rodkey , K. K. Briggs , and J. J. Rodrigo , “Die Technik Der Mikrofrakturierung Zur Behandlung Von Kompletten Knorpeldefekten Im Kniegelenk [the Microfracture Technic in the Management of Complete Cartilage Defects in the Knee Joint],” Der Orthopäde 28, no. 1 (1999): 26–32, 10.1007/s001320050318. 10081041

[14] E. Inderhaug and E. Solheim , “Osteochondral Autograft Transplant (Mosaicplasty) for Knee Articular Cartilage Defects,” JBJS Essential Surgical Techniques 9, no. 4 (2019): e34.1‐2, 10.2106/JBJS.ST.18.00113. PMC6974309 32051778

[15] M. Brittberg , A. Lindahl , A. Nilsson , C. Ohlsson , O. Isaksson , and L. Peterson , “Treatment of Deep Cartilage Defects in the Knee With Autologous Chondrocyte Transplantation,” New England Journal of Medicine 331, no. 14 (1994): 889–895, 10.1056/NEJM199410063311401. 8078550

[16] K. S. Klein , C. M. Aland , H. C. Kim , J. Eisele , and P. Saidi , “Long Term Follow‐Up of Arthroscopic Synovectomy for Chronic Hemophilic Synovitis,” Arthroscopy 3, no. 4 (1987): 231–236, 10.1016/s0749-8063(87)80116-0. 3689521

[17] T. J. Limbird and S. C. Dennis , “Synovectomy and Continuous Passive Motion (CPM) in Hemophiliac Patients,” Arthroscopy 3, no. 2 (1987): 74–79, 10.1016/s0749-8063(87)80019-1. 3606769

[18] L. Poggini , A. Chistolini , G. Mariani , and P. P. Mariani , “Arthroscopic Synovectomy in the Treatment of Haemophilic Arthropathy: Preliminary Results in Eight Patients,” Italian Journal of Orthopaedics and Traumatology 15, no. 4 (1989): 457–461. 2634638

[19] S. J. Triantafyllou , G. A. Hanks , J. A. Handal , and R. B. Greer, 3rd , “Open and Arthroscopic Synovectomy in Hemophilic Arthropathy of the Knee,” Clinical Orthopaedics and Related Research 283 (1992): 196–204. 1395245

[20] J. M. Journeycake , K. L. Miller , A. M. Anderson , G. R. Buchanan , and M. Finnegan , “Arthroscopic Synovectomy in Children and Adolescents With Hemophilia,” Journal of Pediatric Hematology/Oncology 25, no. 9 (2003): 726–731, 10.1097/00043426-200309000-00010. 12972809

[21] A. L. Dunn , M. T. Busch , J. B. Wyly , K. M. Sullivan , and T. C. Abshire , “Arthroscopic Synovectomy for Hemophilic Joint Disease in a Pediatric Population,” Journal of Pediatric Orthopedics 24 (2004): 414–426, 10.1097/00004694-200407000-00013. 15205625

[22] K. H. Yoon , D. K. Bae , H. S. Kim , and S. J. Song , “Arthroscopic Synovectomy in Haemophilic Arthropathy of the Knee,” International Orthopaedics 29, no. 5 (2005): 296–300, 10.1007/s00264-005-0666-2. 16082543 PMC3456637

[23] A. M. de Almeida , M. U. de Rezende , F. G. Cordeiro , et al., “Arthroscopic Partial Anterior Synovectomy of the Knee on Patients With Haemophilia,” Knee Surgery, Sports Traumatology, Arthroscopy 23, no. 3 (2015): 785–791, 10.1007/s00167-013-2706-6. 25839071

[24] E. C. Rodriguez‐Merchan and P. Gomez‐Cardero , “Arthroscopic Knee Debridement Can Delay Total Knee Replacement in Painful Moderate Haemophilic Arthropathy of the Knee in Adult Patients,” Blood Coagulation & Fibrinolysis 27, no. 6 (2016): 645–647, 10.1097/MBC.0000000000000443. 26575489

[25] R. Buda , M. Cavallo , F. Castagnini , et al., “Treatment of Hemophilic Ankle Arthropathy With One‐Step Arthroscopic Bone Marrow‐Derived Cells Transplantation,” Cartilage 6, no. 3 (2015): 150–155, 10.1177/1947603515574286. 26175860 PMC4481389

[26] J. D. Wiedel , “Arthroscopic Synovectomy for Chronic Hemophilic Synovitis of the Knee,” Arthroscopy 1, no. 3 (1985): 205–209, 10.1016/s0749-8063(85)80013-x. 4096772

[27] J. D. Wiedel , “Arthroscopy of the Knee in Hemophilia,” Progress in Clinical and Biological Research 324 (1990): 231–239. 2308968

[28] Z. Salis , B. Gallego , T. V. Nguyen , and A. Sainsbury , “Association of Decrease in Body Mass Index With Reduced Incidence and Progression of the Structural Defects of Knee Osteoarthritis: A Prospective Multi‐Cohort Study,” Arthritis & Rhematology 75, no. 4 (2023): 533–543, 10.1002/art.42307. 35974435

[29] D. Blalock , A. Miller , M. Tilley , and J. Wang , “Joint Instability and Osteoarthritis,” Clinical Medicine Insights: Arthritis and Musculoskeletal Disorders 19 (2015): 15–23, 10.4137/CMAMD.S22147. PMC4337591 25741184

[30] T. O. McKinley , Y. Tochigi , M. J. Rudert , and T. D. Brown , “The Effect of Incongruity and Instability on Contact Stress Directional Gradients in Human Cadaveric Ankles,” Osteoarthritis and Cartilage 16, no. 11 (2008): 1363–1369, 10.1016/j.joca.2008.04.005. 18511308 PMC2592197

[31] Y. Wanezaki , A. Suzuki , Y. Takakubo , et al., “Lower Limb Alignment in Healthy Japanese Adults,” Journal of Orthopaedic Science 28, no. 1 (2023): 200–203, 10.1016/j.jos.2021.10.016. 34815138

[32] R. Kura , E. Sasaki , E. Sato , et al., “Normative Values of Radiographic Parameters in Coronal Plane Lower Limb Alignment in a General Japanese Population: A Cross‐Sectional Study in the Iwaki Cohort,” Journal of Experimental Orthopaedics 12, no. 2 (2025): e70207, 10.1002/jeo2.70207. 40170701 PMC11959492

[33] L. Sharma , J. Song , D. T. Felson , S. Cahue , E. Shamiyeh , and D. D. Dunlop , “The Role of Knee Alignment in Disease Progression and Functional Decline in Knee Osteoarthritis,” Journal of the American Medical Association 286, no. 2 (2001): 188–195, 10.1001/jama.286.2.188. 11448282

